# Social Trust and Anti-immigrant Attitudes in Europe: A Longitudinal Multi-Level Analysis

**DOI:** 10.3389/fsoc.2021.604884

**Published:** 2021-04-09

**Authors:** Jeffrey Mitchell

**Affiliations:** Department of Sociology, Umeå University, Umeå, Sweden

**Keywords:** immigration attitudes, social trust, social change, group threat theory, longitudial analysis

## Abstract

Research investigating how social conditions influence attitudes about immigrants has focused primarily on demographic and economic factors as potential threat inducing contexts that lead to anti-immigrant sentiment. However, the empirical evidence supporting this link is mixed, while social cohesion indicators such as the influence of social trust, have largely been left unexamined. This article uses the European Social Survey (2002–2016) to test how differences in social trust, both within and between countries influence attitudes about immigrants. Results from longitudinal analyses show that countries with higher levels of social trust have more favorable attitudes toward immigrants, and while changes in social trust over time are small, they result in comparably large changes in anti-immigrant attitudes, even when controlling for other social factors. These results are robust across different model specifications and data sources.

## Introduction

There has been a considerable amount of literature dedicated to attitudes about immigrants, often looking for relationships between different social contexts and differences in attitudes in societies. Much of this attention is guided by what has grown to be known as group threat or realistic conflict theory. In an attempt to tie contexts to attitudes, scholars have relied heavily on Blumer's seminal essay *Race Prejudice as a Response to Group Position* (1958), which argues that prejudice is a result of feelings of threat posed by some out-group. However, in the 60 years since its writing the evidence supporting this theory is in a state of disarray. Typically analyzing the importance of how contexts such as proportions of foreign born populations, or economic conditions influence attitudes about immigrants, researchers have found mixed results (for reviews see: Ceobanu and Escandell, 2010; Fussell, 2014; Pottie-Sherman and Wilkes, 2017; Kaufmann and Goodwin, 2018). Instead, the evidence supporting the link between contexts and prejudicial attitudes appear either when analyzing change (Meuleman et al., 2009; Lancee and Pardos-Prado, 2013) or in what can be called “non-material contexts” such as media, political and religious environments. Still, few articles have analyzed how changes in non-material contexts might increase threat, and as the theory suggests, prejudice. This article advocates for a return to Blumer's writing on prejudice, and makes a case for a dynamic interpretation of the theory that focuses on how non-material contexts such as social trust, and particularly changes in those contexts, influence prejudicial attitudes toward immigrants in society.

Surprisingly, even though social trust is common in social scientific research, its relationship to prejudice and specifically anti-immigrant attitudes is mostly unexamined. When scholars have considered trust as it relates to immigration, it is commonly operationalized as a dependent variable, and even subject to the same material contextual predictors testing if proportions of immigrants and economic condition are associated with individual or country level trust (Delhey and Newton, 2005; Stolle et al., 2008; Ziller et al., 2018). However, these results are also mixed. For example, Fairbrother and Martin (2013) show that levels of inequality matter for trust between but not within societies longitudinally. Dinesen and Sønderskov (2015) argue that ethnic diversity at the neighborhood level undermine individuals' social trust, while others argue that this effect disappears when models include metrics for intergroup contact (Koopmans and Veit, 2014; Mckenna et al., 2018), and McLaren (2017) shows the relationship between levels of immigrants and social trust is a function of individuals' level of national identity.

Yet, as it is argued in the social cohesion literature, social trust should be seen as both an individual's perception of the world around them, as well as state of affairs in a society that should be analyzed at the level of the nation state (Chan et al., 2006; Schiefer and van der Noll, 2017). Indeed, it is becoming more common to apply trust at the country level to test associations with other aspects of society including environmental attitudes (Fairbrother, 2016), support for the welfare state (Edlund, 2006), and health (Kim et al., 2011). Both the importance that the threat literature places on non-material contexts, and the import role societal trust plays in relation to other aspects of society leads to the question: Are more trusting societies more welcoming toward immigrants? Also, do changes in the level of trust within societies translate to more or less friendly attitudes toward immigrants? This study addresses these questions by analyzing macro-level generalized trust indicators both within and between societies using multi-level analysis of eight waves of the European Social Survey between 2002 and 2016. To ensure the findings are robust, supplemental country-country analyses testing historic trust levels on contemporary immigration sentiment metrics using the European and World Values Surveys are also reported.

## Group Threat, the Case for Non-material Contexts, and Change

The previous sociological literature about how context influences attitudes toward immigrants has focused on testing aspects of group threat theory, which argues that in-groups feel they have exclusive proprietary claims to aspects of society (Blumer, 1958) and when those proprietary claims are under threat, prejudicial attitudes toward out groups are the result. While this theory has been elaborated upon to include, for example, different types of threat in different conditions (Blalock, 1973), contemporarily what may be its largest contribution is the importance it places on prejudice as a collective process. This, as Bobo notes, was intended to “shift analytical attention away from processes internal to the individual while still recognizing that individual prejudice was a powerful social force,” the strength being that it is “a general attitude orientation involving normative ideas about where one's own group should stand in the social order vis-à-vis an out-group” (Bobo, 1999, p. 448–449).

This has led researchers to pursue a multilevel analytical framework, analyzing whether material indicators such as economic stress and high proportions of out-group populations might provide evidence of higher levels of prejudicial attitudes. Quillian's study 1995 argued that collective threat, manifest as out-group prejudice, was a function of these two factors finding evidence of this relationship in 12 European countries. Since, many scholars have tried to replicate these findings across a multitude of different contexts with mixed results. However, in their meta-analysis of studies using proportions of immigrants as a threat inducing contextual factor, Pottie-Sherman and Wilkes (2017) caution researchers and policy makers attempting to make an empirical or conceptual link between out-group size and attitudes. They write, “While some find a positive relationship between out group population size and attitudes, other's find a negative relationship or not relationship at all” (p. 243). It could be that measuring population size is too coarse of a metric to be applied to Blumer's threat condition, especially since as immigration populations accumulate over time there are increased chances of inter-group contact which has been shown to reduce out-group prejudice (Allport, 1954; Pettigrew, 1998; Pettigrew and Tropp, 2006). These two processes occurring in tandem may be confounding population based studies testing threat (Schlueter and Scheepers, 2010).

Quillian's study also found evidence that economic conditions that induce threat in societies are related to more anti-immigrant attitudes. Others have supported this claim, finding a negative relationship with unemployment rates and decreases in GDP (Meuleman et al., 2009; Billiet et al., 2014) Still, similar to the research on proportions of immigrants, the empirical evidence that supports the claim that conditions of economic threat drive prejudice is mixed, with other studies finding no relationship (Hjerm, 2007; Sides and Citrin, 2007). Clearly, the literature supporting the link between material threat conditions in societies and anti-immigrant attitudes is tenuous. In fact, in a review of the literature Hainmueller and Hopkins went so far as to call it a “zombie theory” 2014.

The mixed results in the empirical findings illustrated by reviews of the literature suggests a reckoning for group threat theory. In this case, it is productive to return to the original text for guidance for what contextual factors should be important in the group processes that might induce perceptions of threat. In it, Blumer lists four types of feelings that accompany group prejudice. “They are (1) a feeling of superiority, (2) a feeling that the subordinate race is intrinsically different and alien, (3) a feeling of proprietary claim to certain areas of privilege and advantage, and (4) a fear and suspicion that the subordinate race harbors designs on the prerogatives of the dominate race” (1958, p. 4). It is true, that researchers have linked individual level attributes to each of the four traits that Blumer lists. For example, the social dominance orientation research addresses Blumer's association of feelings of superiority. It suggests that societies minimize group conflict creating ideologies that promote the superiority of one group over another. This is done in part through the creation of legitimization myths that justify these feelings of superiority (Pratto et al., 1994). Furthermore, studies about cultural distance lend support to the idea that outgroups that are perceived as “intrinsically different” spurn more prejudice than do outgroups that are more similar to the host population. In support of this idea Bohman and Hjerm (2014) found that more religiously homogeneous societies were more immigrant averse. Also, while prejudice is sometimes said to be related to non-western immigrants, Manevska and Achterberg (2013) find that this relationship is dependent on individual's social dominance orientation. Both of these areas of research focus on individual traits or perceptions, and where there is support for proprietary claims driving prejudice it also lies in people's perceptions of the economy or feelings about immigration levels, that may have nothing to do with the material conditions in their environments (Nadeau et al., 1993; Kuntz et al., 2017).

In contrast, non-material contexts have been more consistently linked to prejudicial attitudes. Media environments, religious, and political contexts have all been shown to have significant and substantial influences on attitudes about immigrants in both comparative and longitudinal studies. Hopkins (2010) analyzed a variety of non-material indicators that might influence anti-immigrant attitudes and found that both local-level hostile political policies, and national level political rhetoric in the post September 11th era had negative effects about people's attitudes toward immigrants in the United States. In the European context, political parties that incorporated anti-immigrant rhetoric negatively influenced people's attitudes about immigrants especially if they identified with that political party (Bohman, 2011). Longitudinal studies in Germany (Czymara and Dochow, 2018), Denmark and the Netherlands (Klingeren et al., 2015) show that media saliency of the issue of immigration tends to result in more negative attitudes about immigrants, but that positive coverage of the issue can positively influence them as well. The evidence these studies provide are in line with the theory, since Blumer argues that influential people and media agents help shape whether the in-group members should view their position as threatened in relation to the sub-ordinate out groups. This helps to explain how the process unfolds in relation to contexts under which the issue of immigration becomes culturally and politically salient.

However, it arguably does not help to explain why people and societies are vulnerable to anti-immigrant prejudices. The final requisite Blumer theorizes is “a fear and suspicion that the subordinate race harbors designs on the prerogatives of the dominate race” (4). In themselves, ideas that another group is intrinsically different or inferior, do not pose a threat to the proprietary claims of in-group members unless there is a fear that the out-group has the idea that they may be able to disrupt those proprietary claims and assert them as their own. As has been noted, the group threat literature has primarily focused on linking prejudicial attitudes to material resources that, under threat of deprivation by an out group should trigger feelings of threat. However, since the empirical evidence supporting this link only appears in individuals' feelings about, rather than objective levels of material conditions; then establishing why there is a miss-match between objective vs. perceived conditions is important. It is possible that environments of social (dis)trust engender an environment that induce threat, and in these environments, group based identities become salient and in-group members begin to believe that out groups are (1) inferior, (2) intrinsically different, and that they harbor designs against the proprietary claims of the in-group (3 and 4). In contrast, trusting environments would insulate people from perceiving that others harbor designs against them and prevent their perceived deprivation from being exaggerated, and thus not perceive outgroups as threatening.

### Social Trust

Sociologically speaking social trust is difficult to define. In their writing about the importance of social trust as a dimension of social cohesion Chan et al. explain that it is a quasi-tautology since “it is virtually impossible to conceive of a situation in which we say people are sticking together even though they refuse to trust or cooperate with each other” (2006, p. 289). In other words a certain degree of social trust is a requirement for a cohesive society since, it relies on the expectation that other people's behavior is predictable and that they are led by positive intentions (Schiefer and van der Noll, 2017). However, social trust exists in multiple levels. At the individual level, Freitag and Traunmüller (2009) argue that people harbor different “spheres of trust,” one sphere encompasses individuals' close personal and familial ties, and another generalized trust that is extended to a generalized other that is not personally known. Researchers have wondered whether individuals are responding about feelings toward their own group when asked about whether, generally speaking they feel that they can trust others, however recent research suggests when thinking about the “generalized other,” high trustors include immigrants into this group (van der Liden et al., 2017). As other scholars have pointed out, a possible mechanism for this is the “social intelligence” that high-trusting individuals, who are more likely have a diverse set of experiences, receive in their lives. Over time their experiences inform their judgments about who should and shouldn't be trusted and they are able to rely less on heuristics based on ethnic or cultural stereotypes (Yamagishi, 2001; Herreros and Criado, 2009). In contrast to the type of “bonding” social capital characterized by the inner sphere of close contacts, this social intelligence is a “bridging” social capital that extends to wider ranges of the generalized other. Those with higher levels of bridging social capital, then would not associate out-group members as threatening (Putnam, 2000; Chu and Yang, 2019).

As a collective attribute at the group level or country level, Lewis and Weigert argue that “trust is applicable to the relations among people rather than to their psychological states taken individually. Therefore, we may say that trust exists in a social system insofar as the members of that system act according to and are secure in the expected futures constituted by the presence of each other or their symbolic representations” (Lewis and Weigert, 1985, p. 968). Similarly, as an aspect of social cohesion Chan et al. (2006) argue that social trust should be analyzed at the level of the nation state, since it is an aspect of the political community of each society. In this way, social trust is conceptualized as a state of affairs that guides the citizens of a country as they navigate the economic, institutional, and private spheres of their lives. This has been tested in economic terms with evidence that trusting societies spur economic growth through the lowering of transaction costs (Fukuyama, 1995). This idea, that higher levels of social trust grease the gears of society, has also been found to be important for support of welfare state policies (Edlund, 2006), support for state intervention in environmental policies (Fairbrother, 2016), and health outcomes (Kim et al., 2011).

Social trust is key aspect of the milieu where the group processes described by Blumer take place. I argue that as groups define their position in relation to one another the environment of social trust should be a predictor about whether that collective process of definition results in feelings of threat that results in prejudice. In other words, in low trust environments in-group members “fear and suspicion that the subordinate race harbors designs on the prerogatives of the dominant race” should be able to flourish more than in high-trust environments. There is limited empirical evidence supporting the link between generalized trust and attitudes about immigrants. For example, Herreros and Criado (2009) found that generalized social trust at the individual level is linked to lower levels of anti-immigrant attitudes using the 2002 round of the ESS, even controlling for contextual level threat indicators at the country level. This finding is supported with evidence from the United States, however the link between trust and prejudice differs among different ethnic and racial groups (Chu and Yang, 2019). Additionally, applying trust to the macro level Manevska and Achterberg (2013) found that controlling for many different contextual level variables in the ESS, only social trust remained statistically significant. Finally, using the 2008 wave of the European Values Survey, Ekici and Yucel (2015) report that individual and contextual level social trust were associated with lower level of religious and racial prejudice. While these findings are encouraging, to the author's knowledge, no study has yet to test the relationship between social trust and attitudes about immigrants longitudinally while also modeling the longitudinal effects of material conditions. Understanding the importance of different levels of social trust, it is hypothesized that more trusting individuals will extend their sphere of trust to immigrants.

Hypothesis 1: Individual-level social trust will have a negative relationship to anti-immigrant attitudes

Applying the same idea to contextual environments of social trust, it is hypothesized that in societies with high levels of social trust will be prejudice averse, insulating their citizens from the type of inter-group relations that induce threat feelings.

Hypothesis 2: Country-level social trust will have a negative relationship to anti-immigrant attitudes

### Change Matters

Blumer however, argues that prejudice is “fundamentally a *collective process*” (1958, p. 3, emphasis in original) where the dominant group understands its social position in relation to the subordinate group. Therefore, some of the inconsistent findings identified in the literature might be because studies are applying static empirical analysis to an inherently dynamic relationship. Recently, scholars have focused more on an interpretation of group threat theory as a dynamic process that accounts for change. This approach posits that individual changes in prejudicial attitudes are a reaction to changes in the contextual conditions specified by group threat theory. In this case, changes in conditions make salient issues of intergroup conflict, so that rising or falling proportions of outgroup members overtime should correspond to changes in attitudes. Meuleman et al. summarized this dynamic interpretation to the theory arguing that “actual competition could remain constant at a high level without affecting outgroup attitudes. It is only when sudden changes in minority group size or economic conditions occur that outgroup attitudes evolve.” (2009, p. 354). This could be due to the fact that, as Blumer noted, prejudice is a response to threat that individuals perceive toward their in-group vis-à-vis another group. This way the issue of large proportions of immigrants in an individual's area may not translate to prejudicial attitudes as long as conditions are stable, because there is not a perceived threat of a shift in inter-group power dynamics. However, once the issue is made salient by changes over time, feelings of threat as described in this interpretation of the theory manifest and result in a change in attitudes toward outgroups. This approach has received limited, but encouraging support in empirical tests across a variety of European countries (Meuleman et al., 2009; Lancee and Pardos-Prado, 2013; Czymara, 2020), the United States (Hopkins, 2010) and in different age groups (Coenders and Scheepers, 2008; Mitchell, 2019).

Still, the changing contexts described here have all been material, referring to changes in either demographic or economic conditions. Applying this idea to changes in social trust has not yet been empirically tested, however similar theoretical arguments can be made in favor of this approach. For example, changes in other non-material or cultural contexts such as media environments have been linked to changes in attitudes (Klingeren et al., 2015; Czymara and Dochow, 2018). While it is true that social trust is relatively stable over time, it is possible that changes in social trust are linked to changes in attitudes about immigrants. When societal social trust begins to drop, the “state of affairs” becomes one where people are unable to predict that others will react in good faith, increasing the probability that the sphere of trust will be contracted to exclude immigrants and contribute to the fear that they harbor designs against the prerogatives of the dominant group. Taking the dynamic approach to group threat theory, and informed by the importance of non-material contexts, I hypothesis that changes in social trust will have an impact on in-group members attitudes toward immigrants:

Hypothesis 3: Over time reductions in trust within countries will result in higher anti-immigrant attitudes over time

## Data and Methods

Data for the dependent variable and main independent variable come from eight rounds of the European Social Survey (ESS) from 2002 to 2016. The ESS is a cross-national survey with representative samples of 34 countries, all of them are included in this study. While only 15 countries are included in each of the 8 waves, 34 of them are included in at least two of the waves, resulting in 198 country-years with 268,995 respondents^1^. To analyze the attitudes of the in-groups the sample was restricted to respondents that were born in the response country.

### Dependent Variable

Three items were included in each of the eight waves of the ESS to measure attitudes about immigrants^2^. While the ESS includes periodic modules with more detailed questions about attitudes toward immigrants in 2002 and 2016, a key part of this study is the incorporation of how changes in contexts influence changes in attitudes toward immigrants, so the incorporation of the most waves possible offers the best possibility to gain insight about those changes. Questions about how respondents feel about immigrants that are in their country are measured on a 0–10 point scale where low values represent negative responses and high values represent positive responses. The questions are:

- “Would you say it is generally bad or good for [country]'s economy that people come to live here from other countries?” (0= “Bad for the economy,” 10 = “Good for the economy”).
- “Would you say that [country]'s cultural life is generally undermined or enriched by people coming to live here from other countries?” (0 = “Cultural life undermined,” 10= “Cultural life enriched”). And
- “Is [country] made a worse or a better place to live by people coming to live here from other countries?,” (0= “Worse place to live,” 10 = “Better place to live”).

**Figure 1:**
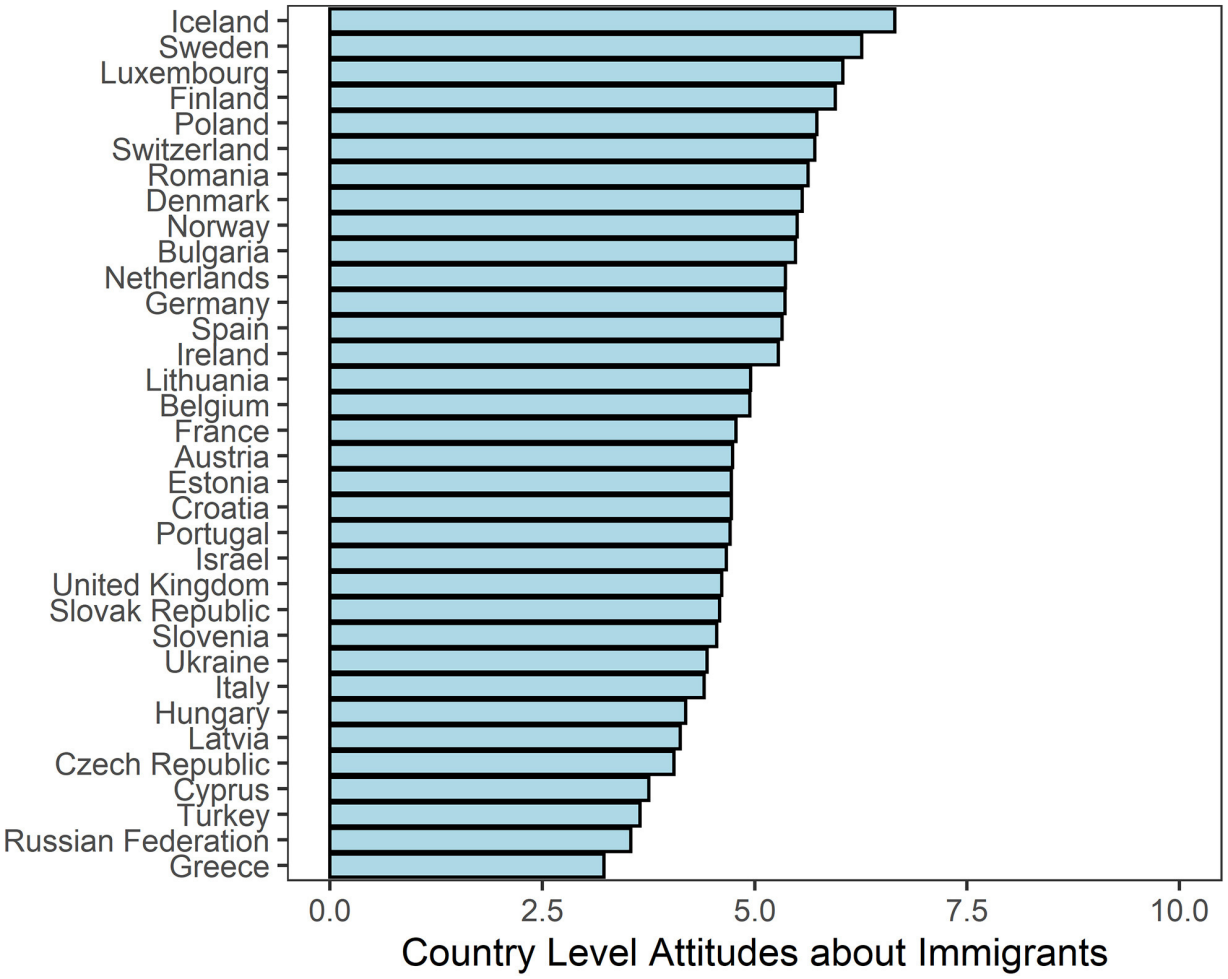
Attitudes about immigrants, averaged across all waves.

An index was created by averaging responses to each of the three questions. This index concerning attitudes about immigrants has a high inter-item average correlation (0.65) and inter-item reliability (Chronbach's alpha = 0.85). Country level averages of the index are depicted in Figure 1. Descriptive statistics for average attitudes about immigrants with their standard deviations in countries across survey years are in available in the Appendix.

### Independent Individual and Contextual Variables

The main independent variable measures generalized trust. At the individual level, trust is measured through the question, “generally speaking, would you say that most people can be trusted, or that you can't be too careful in dealing with people?” On a 10 point scale (0= “You cant be too careful,” 10= “most people can be trusted”). To isolate individuals' trust levels in a way that is relative to the respondents' country, responses were centered against the average level of generalized trust in that country at that time. This means that, the variable included in the analysis is the level of difference people say that they can trust others, compared to others in their country during the survey wave.

**Figure 2:**
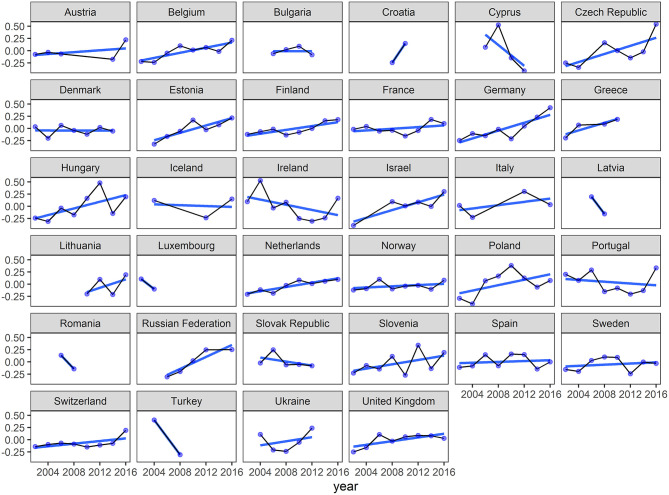
Within-country social trust over time.

To create a country level contextual trust variable, this study employed a series of aggregation and centering methods to parse out the effects of the differences in trust between countries, vs. the changes in levels of trust within countries (Fairbrother, 2014). First, to establish a time invariant country level trust measure to assess the differences between countries, responses were aggregated by country and averaged across all response waves. Next, to assess the differences within countries over time, the average value of trust in each country-year was subtracted from the time-invariant level of trust in that country. This way, the within-country trust measurement is the degree to which that wave deviates from the time invariant, between-country average. Country averages are also included in Appendix, and Figure 2 shows the within-country measurements of social trust for each wave of the ESS.

While using the 10 point ESS scale is advantageous for this study since it is included in all eight rounds, there is some evidence that using this scale biases responders to report higher levels of trust than using other scales (Bekkers and Sandberg, 2019). While within country changes in social trust should not be influenced by this bias since the ESS uses the same scale in each wave, it is possible that the between country analysis is influenced. To address this issue, and to incorporate a historical trust perspective, two additional datasets were brought in for supplemental analyses. A binary trust indicator from the European Values Survey in 1999 was aggregated at the country level to represent a proportion of respondents that respond “most people can be trusted.” This country level trust variable was merged with the 2016 ESS wave to test the relationship between historic levels of trust and attitudes about immigrants. To test if this relationship is Europe specific, additional analysis of the World Values Survey with the same binary variable for trust for the 1995–96, and the 2010–12 wave variable measuring respondent's willingness to live next to immigrants were used.

To further test the for the importance of non-material contexts, and to control for a possible confounder of political environments that might influence both societal trust and attitudes about immigrants, country-level data from the Manifesto Project (Volkens et al., 2020) is included in the analysis. The MP data is a quantitative content analysis of a corpus of party manifestos. Following Bohman (2011), the analysis incorporates a national way of life (NWOL, *per601*) indicator, which is a measurement of how often political parties make positive appeals to patriotism and nationalism, support for established national ideas, and protection of the state from subversion. Each country-year observation is the average value of NWOL articulations, standardized for the number of parties and their representation in parliament in the nearest election before the ESS round.

To test the classical approach of threat to proprietary claims of group threat theory, metrics for proportions of foreign born people and gross domestic product (GDP) were taken from the World Development Indicators database compiled by the World Bank. While data for GDP was available for all years, proportions of foreign born people were only available every 5 years (2000, 2005, 2010, and 2015). Linear interpolation imputed values between the provided years in the WDI data for proportion foreign born and was merged into the ESS along with the logGDP for each wave. To assess how the differences in levels of political contexts measured with the NWOL indicator, GDP, and foreign born populations between countries, vs. the changes that are occurring within them the same within-between centering process that was constructed with the social trust context variable was used.

In addition to the individual level social trust variable a series of other individual level variables were included in the analysis. Age was coded as a categorical variable labeled “young” (25 and younger), “old”(65 and older), and 26–64 (ref.). Controlling for individual level socio-economic differences comparable across countries a variable for education separating people with university education (1) an without (0) is included, as well as gender (male ref.) and a self reported house hold income metric about whether people feel like they are living comfortably on their present income. On a 4 point scale, 1= “living comfortably on present income,” 4= “very difficult on present income.” Personal political orientation in the form of a left (0)-right (10) scale is also included.

### Analytic Methods

Due to the structure of the data, a multi-level modeling approach that nests respondents inside of country-years and counties was employed for this study. This allows for both the estimation of the relationship between contextual variables of interest and anti-immigrant attitudes both cross-sectionally and longitudinally. Since the responses to the surveys are in part dependent on the groups that the respondents belong, models that accommodate for this country and country-year grouping are better suited for this analysis than models with no grouping structure (Fairbrother, 2014). In the statistical model, for each of the contextual level variables I add the de-meaned value and its mean. The advantage of including the time-varying de-meaned value (within-effects WE), rather than the raw value, is that it controls for time invariant differences between countries (such as welfare state regime type, and immigration policies) by basing the estimates on the variance occurring only within nations over time. The meaned, between country values (between-effects BE) are then included to estimate the time invariant differences between countries (Bell et al., 2019).

The analysis follows a model building approach where Model 1 is an “empty” models for the dependent variable and its nesting structure in the three level model. Model 2 tests the relationship between the individual level independent variables and the dependent variable measuring respondents' attitudes about immigrants. Model 3 adds the country level independent variables, including country level social trust. In the first three models random intercepts are used in estimation, and Model 4 adds random slopes to the estimates in the three- level models^3^.

In the robustness check, models 5–7 are linear models regressing country level attitudes on historic levels of social trust. Each uses different combinations of data sources from the ESS, EVS and WVS for the independent and dependent variables. These models include the interaction effects of historical trust with changes in proportion of foreign born people. While the sample sizes of the country level analyses are small, if historically trusting countries have more friendly attitudes toward immigrants, then high trusting countries should retain better attitudes toward immigrants even after a period of increasing immigration.

## Results

**Figure 3:**
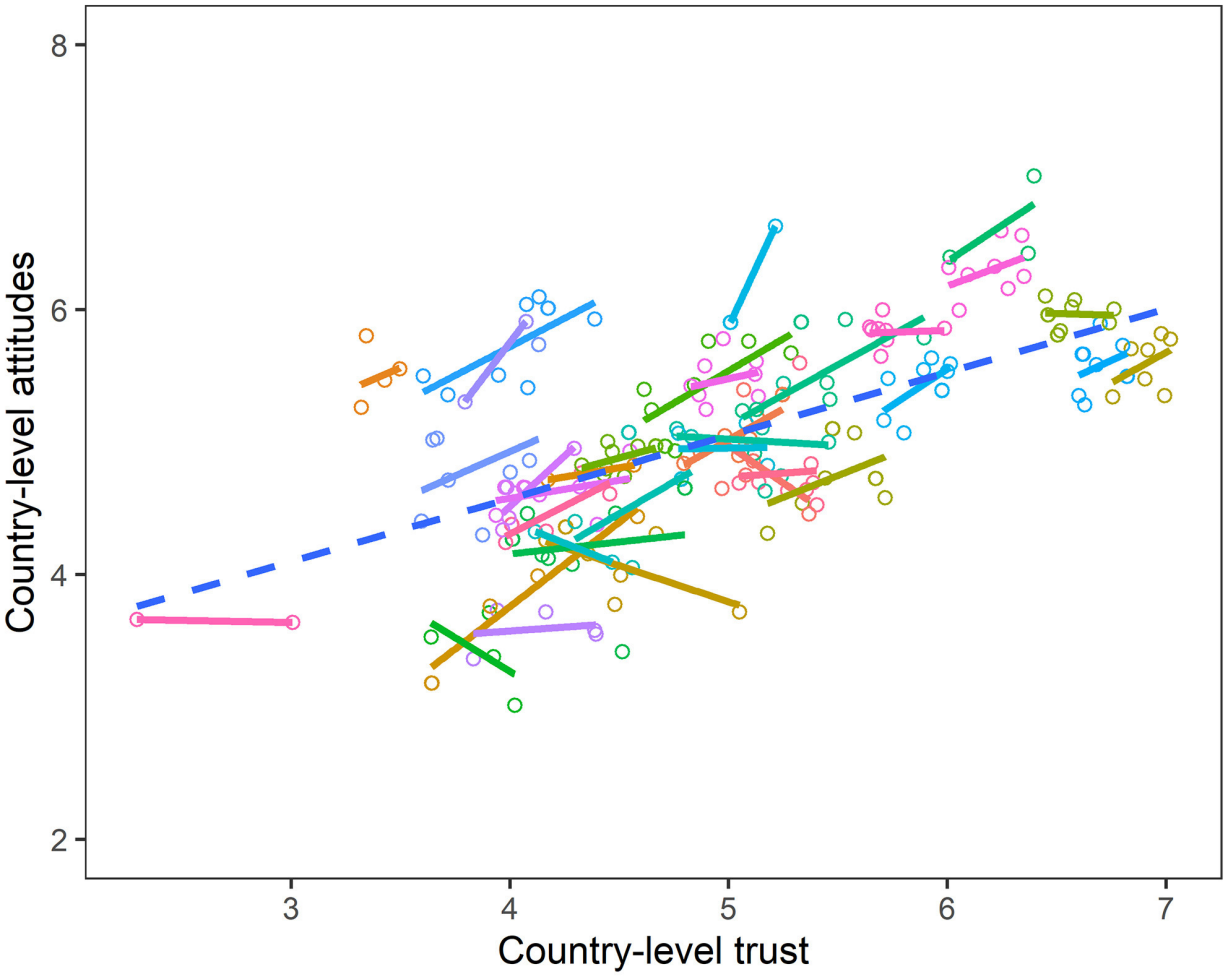
Scatterplot of country-year attitudes about immigrants and social trust.

A visual representation of the relationship between county level attitudes about immigrants and country level social trust is shown in Figure 3. In it, each solid line represents a different country and each dot a country-year. In nearly all the countries there is a positive relationship between levels of social trust and attitudes about immigrants. The dotted line is the correlation over all, showing that this positive relationship is true for the country-year sample as a whole.

Estimates and standard errors from the multi-level models are presented in Table 1. For attitudes about immigrants the estimate for the intercept is 4.93. Model 2 shows that individual level trust is related to more positive attitudes about immigrants (0.20). This is a confirmation of hypothesis one, which is to say that people who are more trusting than their peers in their country, during the time of survey report less prejudicial attitudes toward immigrants. There is also a relationship between age groups of respondents and their attitudes, with people under 25 having more friendly (0.16) attitudes and people older than 65 having less friendly (−0.29) attitudes toward immigrants than the reference category of people between 25 and 65 years of age. Respondents with a university degree have substantially more positive attitudes about immigrants than those without (0.73), similarly those that report lower household income harbor less friendly attitudes that those that report higher (−0.27). Also, people that identify further “right” on the political spectrum have less friendly attitudes toward immigrants (−0.80). In addition to confirming hypothesis 1, the findings from individual level variables support many of the claims found in previous scholarship, for example that individuals that are less vulnerable to threat feelings because of their educational attainment or higher household income harbor less prejudices. Still, controlling for these other individual level factors typically associated with prejudice in the group threat framework, social trust still has a positive relationship with attitudes toward immigrants.

**Table 1:** Multi-level regression models.

	**Model 1**	**Model 2**	**Model 3**	**Model 4**
*Predictors*	*Estimates*	*Estimates*	*Estimates*	*Estimates*
	*std. error*	*std. error*	*std. error*	*std. error*
Intercept	4.93^***^	5.90^***^	11.57^**^	12.27^***^
	−0.14	−0.12	−4.35	−3.67
Indiv. Trust		0.20^***^	0.20^***^	0.20^***^
		0	0	0
25 and under		0.16^***^	0.16^***^	0.16^***^
		−0.01	−0.01	−0.01
65 and older		−0.29^***^	−0.29^***^	−0.29^***^
		−0.01	−0.01	−0.01
University		0.73^***^	0.73^***^	0.73^***^
		−0.01	−0.01	−0.01
Female		−0.03^***^	−0.03^***^	−0.03^***^
		−0.01	−0.01	−0.01
Income		−0.27^***^	−0.27^***^	−0.27^***^
		0	0	0
Left		−0.80^***^	−0.80^***^	−0.80^***^
		−0.02	−0.02	−0.02
WE Trust			0.59^***^	0.60^***^
			−0.12	−0.14
BE Trust			0.44^**^	0.43^***^
			−0.14	−0.12
WE NWOL			−0.01	−0.02
			−0.01	−0.01
BE NWOL			−0.10^**^	−0.11^***^
			−0.04	−0.03
WE logGDP			0.09	−0.02
			−0.36	−0.35
BE logGDP			−0.71	−0.76^*^
			−0.45	−0.38
WE %Foreign Born			0.02	0.03
			−0.02	−0.02
BE %Foreign Born			0.02	0.01
			−0.02	−0.02
ESS Round			−0.03	−0.03
			−0.02	−0.02
**Random effects**				
σ^2^	4.04	3.45	3.45	3.45
τ_00_	0.08 _cntryyr_	0.08 _cntryyr_	0.07 _cntryyr_	0.06 _cntryyr_
	0.62 _cntry_	0.48 _cntry_	0.30 _cntry_	0.30 _cntry_
τ_11_				0.21 _cntry.countrytrust_
ρ_01_				0.86 _cntry_
Countries	34 _cntry_	34 _cntry_	34 _cntry_	34 _cntry_
Country/Year	200 _cntryyr_	198 _cntryyr_	198 _cntryyr_	198 _cntryyr_
Observations	314934	268995	268995	268995
AIC/BIC		1097466/1097582	1097437/1097647	1097429/1097660

* p < 0.05

** p < 0.01

*** *p < 0.001*.

*Dependent variable attitudes about immigrants*.

Turning to the contextual level variables, Model 3 confirms both hypotheses 2 and 3. Average levels of social trust, that is to say the time invariant differences in social trust between countries, is significantly and substantially related to attitudes about immigrants (0.44). Countries with higher levels of social trust, on average translate to more positive attitudes toward immigrants. Similarly, changes in social trust within countries are significantly related to attitudes toward immigrants (0.59). While changes in aggregate social trust are relatively small, they translate to comparably large changes in attitudes about immigrants.

The results also support previous findings about the importance of political contexts in relation to attitudes about immigrants (Bohman, 2011) though only between countries, not within them over time. Countries where NWOL articulations are on average high are less friendly toward immigrants, but over time changes in the salience level of the NWOL indicators is unclear. This might be due to the complicated (and perhaps reciprocal) relationship between parties and their constituents common in the supply and/or demand literature in political science.

**Figure 4:**
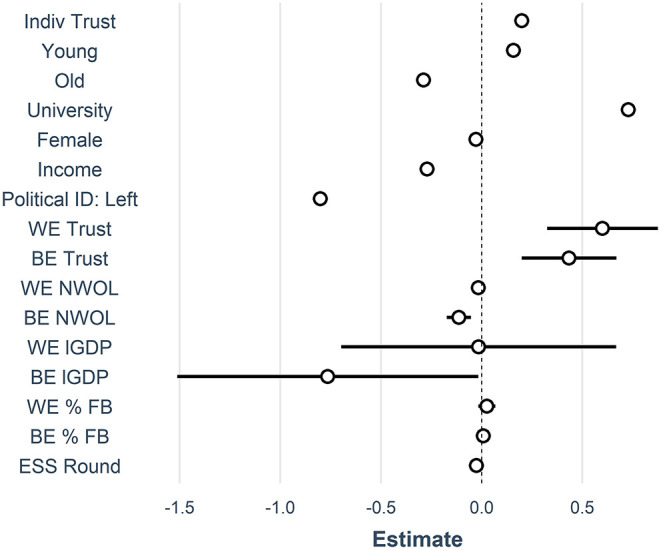
Dot and whisker plot of effect sizes on anti-immigrant attitudes.

In contrast, the contextual level material indicators that have frequently been used in previous research show a complicated relationship with attitudes about immigrants, with estimates are sensitive to model specification and the exclusion of specific countries^4^ (reported in the Appendix). In the full sample reported here, only the between effects of GDP (−0.78) have a substantial relationship to attitudes about immigrants with a large confidence interval (Figure 4), while changes in GDP, as well as the within or between effects of proportions of foreign born people have no relationship with anti-immigrant attitudes. These results lend support to the idea that non-material indicators, like social trust, have a more substantial link than do material indicators both comparatively and over time. It also shows that changes in non-material conditions, as predicted in the dynamic interpretation of group threat theory are important for attitudes about immigrants. Model 4 incorporates random slopes at the country level, but this does not substantially change the results from Model 3. The dot and whisker plot (Figure 4), shows the effect size and standard errors of each of the independent variables centered around the intercept with confidence intervals. Of the contextual variables, both within and between country trust and time invariant levels of GDP do not overlap with zero.

In addition to the evidence provided in Models 1–4, at the country-level, historical trust should be related to levels of prejudice in the future. To check for this, linear regression models at the country-level analyzed the relationship between historical social trust and contemporaneous levels of prejudice controlling for GDP and changes in immigration over the gap in observation times. These analyses used three different combinations of dependent variables and independent variables from three different datasets, the European Values Survey (EVS), European Social Survey (ESS), and the World Values Survey (WVS). Regression tables of these analyses are available in the Appendix, however, results show that countries with historically high social trust are still related to more positive attitudes about immigrants in the future even in small sample sizes. Historical trust from the first wave of the European Social Survey Cumulative File, ESS 1-9 (2020), and from the 1999 wave of the EVS, resulted in statistically significant relationships with future prejudice (European Social Survey Cumulative File, ESS 1-9, 2020). To ensure this relationship is not Europe specific, the same analysis was conducted using WVS trust measurements from 1995 to 96 and attitudes about immigrants in 2010–2012 yielding similar results. Many of the countries included in the three additional analyses experienced notable demographic changes in terms of the number of immigrants, if societal levels of social trust are able to insulate societies from prejudice even in times of demographic change, then high trusting societies that received many immigrants should at least not report *less* positive attitudes toward immigrants. However, as the interaction effects in Figures 5A–C of the Appendix between historic levels of trust and changes in proportions of foreign born show, high trusting countries that saw relatively big demographic changes (1 standard deviation from the average), appear to have *more* positive attitudes than those high trusting countries that did not see such a change. While the sample size of countries is small (18 using ESS and EVS, and 30 using WVS), the findings are significant at the *p* < 0.5 level. Adding confidence to this, the relationship points in the same direction regardless of the data and country samples that were used, suggesting that it is likely that trusting countries are not only better insulated from prejudice than low trusting countries, but perhaps that they are able to become even more friendly in the presence of high immigration through the facilitation of bridging capital (Putnam, 2000).

## Conclusions

The literature examining the effect of country level contexts has been dominated by what has become known as group threat theory, often looking for a link between attitudes and demographic conditions with mixed results. There is a growing body of research that is beginning to look at how non-material contextual conditions might influence these attitudes, yet few studies have examined how social trust might influence attitudes about immigrants in different societies. While a more direct reading of the theory may point to these material contextual indicators, the absence of robust empirical findings over the last 30 years suggest a revisiting of Blumer's original text for a re-reading, and for further guidance. This article proposes a return to theory and an emphasis on Blumer's assertion that group dynamics engender fear, and influence prejudicial attitudes.

The findings here show that generalized trust is linked to lower levels of prejudice toward immigrants both at the individual and country level. At the individual level, this could mean that individuals are extending their circle of trust to include people that are not a part of their in-group, as defined by their membership to a nation-state. This would be consistent with the findings of previous literature. At the country level, group threat theory would argue that environments of trust insulate societies from prejudicial attitudes because the general “state of affairs” where the group dynamics are taking place are ones of reduced fear that out groups, or any group, is harboring prerogatives against the in-group. Furthermore, while country level trust is relatively stable over time, even small changes in social trust correspond to relatively large changes in attitudes about immigrants. This finding is in line with the dynamic approach to group threat theory. It would also appear that countries with historically high levels of trust are able to incorporate comparatively large changes is immigrants and report more positive attitudes toward them. Ensuring that these findings are not an artifact of the data and as a robustness check, historical social trust metrics from different data sources were incorporated in country comparative analyses. Historically trusting countries are on average less prejudicial, even in times of high immigration. This finding is true even outside the European context.

It is possible that the findings here are confounded somewhat by other factors that influence both social trust and prejudice in societies. What comes to mind are social institutions such as robust welfare states that both generate trust and reduce perceptions of threat that might induce prejudice. To further analyze the potential direct and indirect effects social trust environments have on anti-immigrant attitudes, and prejudicial attitudes generally speaking is an avenue for future research. While recognizing this possibility, the relationship shown in this study is still important to note, since finding ways to generate higher levels of social cohesion will have spill-over effects in societies such as the reduced levels of prejudice shown here.

As researchers continue to examine the relationships between contextual influences and anti-immigrant attitudes, the incorporation of social indicators beyond demographic and economic factors that might influence prejudice should receive more consideration. Understanding that these indicators are subject to changes over time must also be kept in mind to account for shifts in attitudes within societies. Fortunately, the recent expansion of datasets that cover more topics over a longer periods of time allow for this type of social inquiry both across societies and within them longitudinally.

## Data Availability Statement

Publicly available datasets were analyzed in this study. This data can be found at: http://www.europeansocialsurvey.org/downloadwizard/. Replication files for data cleaning and analysis can be found here: https://osf.io/7zmhu/.

## Author Contributions

The author confirms being the sole contributor of this work and has approved it for publication.

## Conflict of Interest

The author declares that the research was conducted in the absence of any commercial or financial relationships that could be construed as a potential conflict of interest.

## Footnotes

^1^Albania and Kosovo are excluded for participating in only one wave.

^2^The ESS has a separate battery of questions about attitudes about immigration, gathering information about whether respondents favor immigration from different areas. While these questions are related, the focus of this article is about anti-immigrant sentiment. Still, models testing an immigration index as a dependent variable yielded similar results.

^3^Many different model specifications were tested including the addition of the ESS design weights and attitudes about immigration instead of immigrants as the dependent variable. Each yielded similar results.

^4^The models reported in the Appendix exclude the Russian Federation and the Slovak Republic. In this model, both the WE and BE effects of %FB appear to be related to attitudes about immigrants, but the effect of GDP disappears. In both model specifications the effects of social trust and the NWOL measurements are stable.
