# Supplementary material for: Social Trust and Anti-immigrant Attitudes in Europe: A Longitudinal Multi-Level Analysis
Source: Front Sociol. 2021 Apr 9;6:604884. doi: 10.3389/fsoc.2021.604884 (PMC8064709; doi:10.3389/fsoc.2021.604884)

**Appendix**

**Descriptive statistics of anti-immigrant attitudes and generalized social trust**

|  | Attitudes about immigrants | | Generalized social trust | |  |
| --- | --- | --- | --- | --- | --- |
| Country | *mean* | *s.d.* | *mean* | *s.d.* | *n* |
| Austria | 4.74 | 2.13 | 5.15 | 2.38 | 9751 |
| Belgium | 4.94 | 1.84 | 5.03 | 2.21 | 12760 |
| Bulgaria | 5.48 | 2.41 | 3.41 | 2.59 | 8256 |
| Croatia | 4.73 | 2.2 | 4.42 | 2.42 | 2827 |
| Cyprus | 3.75 | 2.08 | 4.06 | 2.55 | 4071 |
| Czech Republic | 4.05 | 1.96 | 4.5 | 2.42 | 14764 |
| Denmark | 5.56 | 2.03 | 6.96 | 1.97 | 10143 |
| Estonia | 4.73 | 1.97 | 5.5 | 2.15 | 10999 |
| Finland | 5.95 | 1.73 | 6.58 | 1.88 | 15648 |
| France | 4.78 | 2.16 | 4.48 | 2.19 | 13609 |
| Germany | 5.35 | 2.03 | 4.86 | 2.3 | 21236 |
| Greece | 3.22 | 2.08 | 3.83 | 2.39 | 8863 |
| Hungary | 4.19 | 2.01 | 4.32 | 2.38 | 12873 |
| Iceland | 6.65 | 1.76 | 6.25 | 2.2 | 2086 |
| Ireland | 5.28 | 2.23 | 5.37 | 2.42 | 15861 |
| Israel | 4.66 | 2.35 | 5.16 | 2.43 | 10023 |
| Italy | 4.4 | 2.28 | 4.53 | 2.35 | 5954 |
| Latvia | 4.13 | 2.14 | 4.27 | 2.62 | 3459 |
| Lithuania | 4.95 | 1.97 | 4.98 | 2.35 | 7881 |
| Luxembourg | 6.04 | 1.99 | 5.11 | 2.33 | 2216 |
| Netherlands | 5.36 | 1.62 | 5.92 | 2.02 | 13892 |
| Norway | 5.5 | 1.79 | 6.72 | 1.84 | 12014 |
| Poland | 5.73 | 1.88 | 4.01 | 2.41 | 13941 |
| Portugal | 4.71 | 1.89 | 3.8 | 2.34 | 14025 |
| Romania | 5.63 | 2.3 | 3.94 | 2.64 | 4218 |
| Russian Federation | 3.54 | 2.15 | 4.14 | 2.64 | 11728 |
| Slovak Republic | 4.59 | 1.9 | 4.05 | 2.44 | 8545 |
| Slovenia | 4.55 | 2 | 4.21 | 2.48 | 9965 |
| Spain | 5.32 | 1.98 | 4.97 | 2.11 | 14133 |
| Sweden | 6.26 | 1.93 | 6.25 | 2.06 | 12730 |
| Switzerland | 5.71 | 1.76 | 5.79 | 2.11 | 10854 |
| Turkey | 3.65 | 2.56 | 2.6 | 2.69 | 4219 |
| Ukraine | 4.44 | 2.39 | 4.22 | 2.68 | 8899 |
| United Kingdom | 4.61 | 2.23 | 5.3 | 2.18 | 15661 |

**Models showing the relationship between historical trust and attitudes about immigrants**

|  | **ESS 2016/EVS 1999** | | **ESS 2016/ESS 2002** | | **WVS 2010-12/WVS 1995-96** | |
| --- | --- | --- | --- | --- | --- | --- |
| *Predictors* | *Estimates* | *std. Error* | *Estimates* | *std. Error* | *Estimates* | *std. Error* |
| (Intercept) | 5.13 | 0.23 | 5.16 | 0.2 | 0.79 | 0.03 |
| Historical Trust | 2.46 | 1.4 | 0.53 | 0.24 | -0.05 | 0.17 |
| FB Change | 0.08 | 0.07 | 0.01 | 0.06 | 0 | 0.01 |
| Trust X FB Change | 0.83 | 0.75 | 0.04 | 0.09 | 0.18 | 0.07 |
| Observations | 18 | | 19 | | 30 | |
| R2 / R2 adjusted | 0.343 / 0.202 | | 0.359 / 0.230 | | 0.222 / 0.113 | |

**Within and Between model excluding Russian Federation and Slovak Republic**

|  | **Reduced Country Sample** |
| --- | --- |
| *Predictors* | *Estimates* |
| Intercept | 14.33 ^***^ |
|  | -3.88 |
| Indiv. Trust | 0.20 ^***^ |
|  | 0 |
| 25 and under | 0.15 ^***^ |
|  | -0.01 |
| 65 and older | -0.29 ^***^ |
|  | -0.01 |
| University | 0.76 ^***^ |
|  | -0.01 |
| Female | -0.03 ^***^ |
|  | -0.01 |
| Income | -0.27 ^***^ |
|  | -0.01 |
| Left | -0.86 ^***^ |
|  | -0.02 |
| WE Trust | 0.59 ^***^ |
|  | -0.15 |
| BE Trust | 0.43 ^***^ |
|  | -0.12 |
| WE NWOL | 0.01 |
|  | -0.36 |
| BE NWOL | -0.95 ^*^ |
|  | -0.4 |
| WE logGDP | 0.03 |
|  | -0.02 |
| BE logGDP | 0.01 |
|  | -0.02 |
| WE %Foreign Born | -0.03 ^**^ |
|  | -0.01 |
| BE %Foreign Born | -0.14 ^***^ |
|  | -0.04 |
| ESS Round | -0.02 |
|  | -0.02 |
| **Random Effects** | |
| σ^2^ | 3.44 |
| τ_00_ _cntryyr_ | 0.06 |
| τ_00_ _cntry_ | 0.33 |
| τ_11_ _cntry.countrytrustD_ | 0.29 |
| ρ_01_ _cntry_ | 0.89 |
| N _cntry_ | 32 |
| N _cntryyr_ | 188 |
| Observations | 256145 |
| ** p<0.05   ** p<0.01   *** p<0.001* | |
|  | |


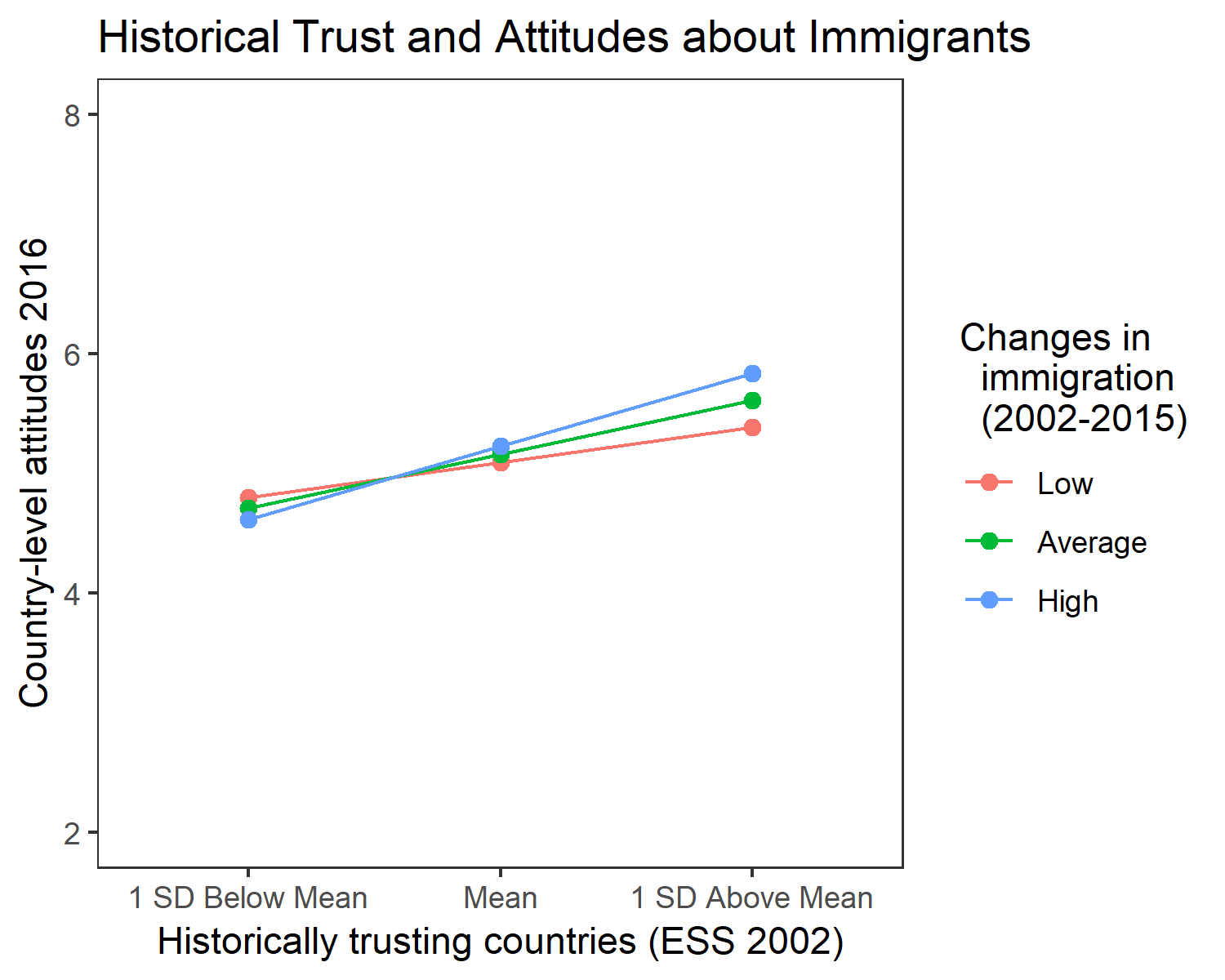

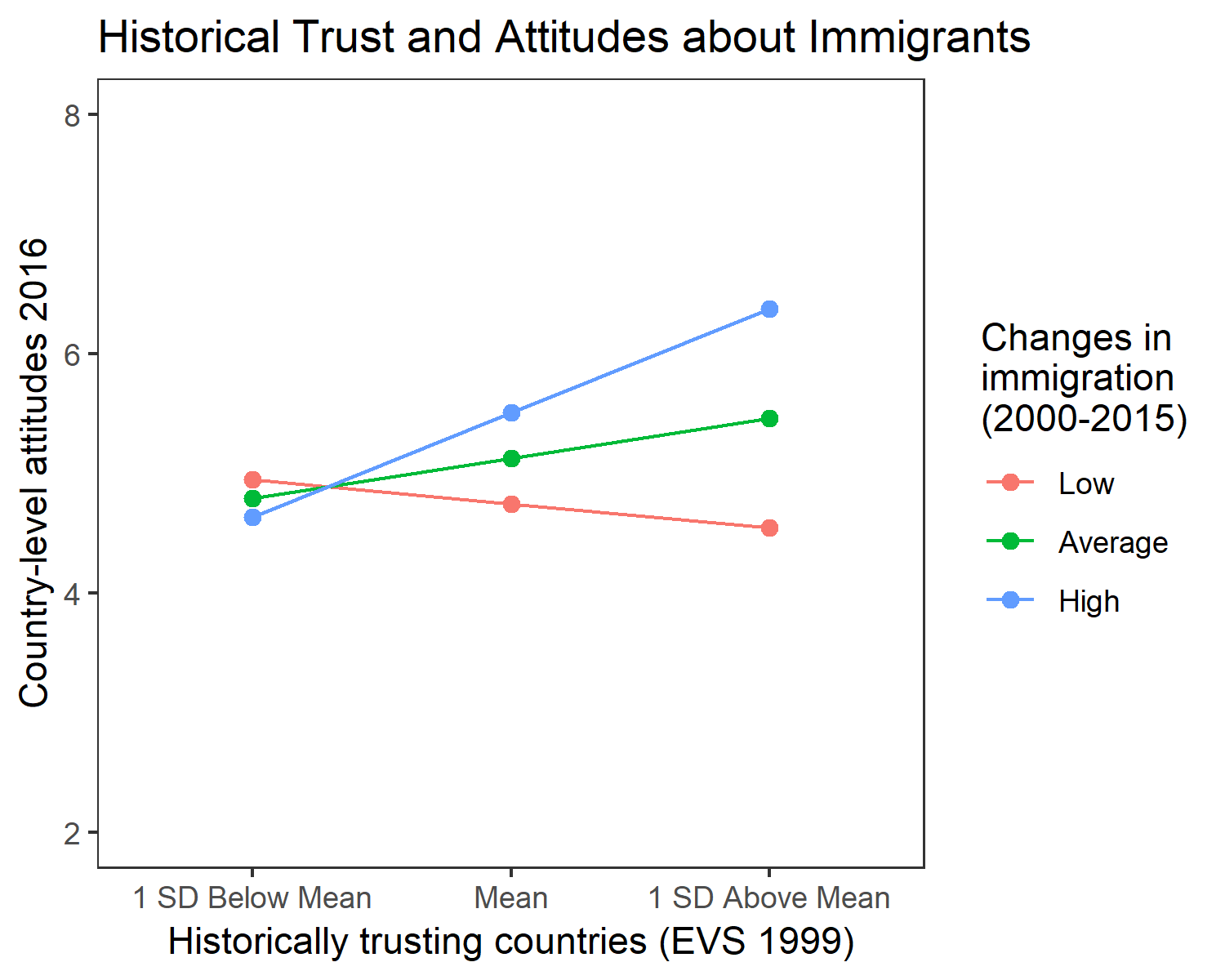

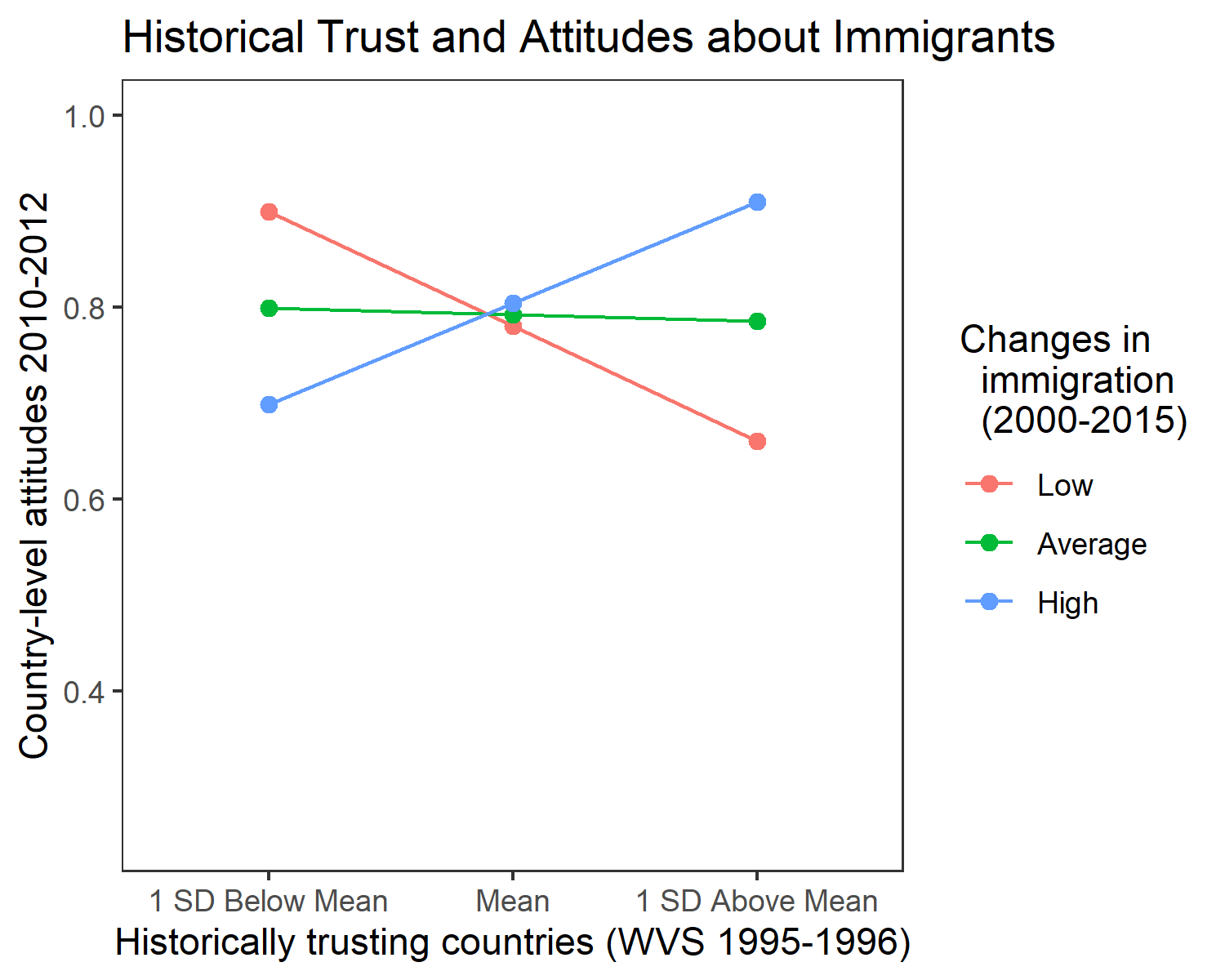

Supplement: Supplementary file 1 [file Data_Sheet_1.docx]
